# The Multidisciplinary Approach to Enhanced Recovery After Surgery in Colorectal Cancer: An Updated Narrative Review

**DOI:** 10.7759/cureus.115176

**Published:** 2026-08-25

**Authors:** Maria Bourazani, Andreas Nikolaos Dafnis, Pavlina-Athanasia Syrogianni

**Affiliations:** 1 Department of Anesthesiology, Hellenic Anticancer Institute, Saint Savvas Hospital, Athens, GRC; 2 Department of Surgical Oncology, Hellenic Anticancer Institute, Saint Savvas Hospital, Athens, GRC

**Keywords:** colorectal cancer surgery, colorectal surgery, ​enhanced recovery after surgery, multidisciplinary care, perioperative care

## Abstract

Enhanced Recovery After Surgery (ERAS) has become the principal framework for perioperative care in elective colorectal cancer surgery, integrating multiple evidence-based interventions across the preoperative, intraoperative, and postoperative phases. Successful implementation depends not only on individual pathway components but on coordinated multidisciplinary delivery involving surgeons, anesthesiologists, nurses, dietitians, physiotherapists, pharmacists, and stoma-care specialists. This updated narrative review synthesizes contemporary evidence on the multidisciplinary implementation of ERAS in elective colorectal cancer surgery, drawing primarily on the 2025 ERAS Society recommendations together with literature published between January 2022 and August 2026, identified through structured searches of PubMed/MEDLINE and Scopus. Thirty-three publications were prioritized for narrative synthesis based on relevance, methodological quality, recency, and contribution to multidisciplinary implementation. The review examines preoperative optimization (patient education, prehabilitation, smoking and alcohol cessation, nutritional and hematologic optimization, bowel preparation), intraoperative-adjacent perioperative measures (fasting and carbohydrate loading, venous thromboembolism prophylaxis), and postoperative recovery components (catheter management, early oral feeding, mobilization, prevention of postoperative ileus, discharge planning), alongside pathway adherence, audit, and implementation science. Contemporary evidence continues to support core ERAS components, although the strength of evidence varies: carbohydrate loading and universal extended thromboprophylaxis, for example, show a less definitive effect on major outcomes than previously assumed, while specific mobilization targets remain pragmatic rather than proven thresholds. Nurses occupy a longitudinal, coordinating role across the entire perioperative trajectory. The findings indicate that ERAS effectiveness in colorectal cancer surgery depends less on individual interventions than on consistent multidisciplinary delivery, sustained pathway adherence, and systematic audit. Future research should prioritize individualized risk stratification, standardized adherence reporting, and rigorous evaluation of multidisciplinary implementation strategies rather than the addition of further isolated interventions.

## Introduction and background

Colorectal cancer (CRC) remains a major global health burden, and surgery continues to represent a cornerstone of curative treatment. Optimization of perioperative care is therefore essential not only for reducing postoperative morbidity and length of hospital stay, but also for supporting functional recovery and continuity of oncological care. Traditional perioperative practices historically included prolonged fasting, routine mechanical bowel preparation, delayed oral intake, liberal intravenous fluids, prolonged bed rest, and extensive use of drains and catheters. Accumulating evidence has challenged several of these practices and supported the transition toward structured, evidence-based perioperative pathways [1].

Enhanced Recovery After Surgery (ERAS) is a multimodal, evidence-based approach to perioperative care designed to attenuate the physiological stress response to surgery, preserve function, reduce complications, and accelerate recovery. Rather than a single intervention, ERAS integrates multiple evidence-based components across the preoperative, intraoperative, and postoperative phases of care [1]. A defining feature of successful implementation is its multidisciplinary nature: surgeons, anesthesiologists, nurses, dietitians, physiotherapists, and pharmacists contribute distinct but complementary expertise, and effectiveness depends on coordinated decision-making, standardized communication, continuity of care, and systematic evaluation of adherence [1].

Nurses have a continuous role across the ERAS pathway, including preoperative education, assessment of patient needs, implementation and monitoring of standardized interventions, pain and symptom assessment, support for early oral intake and mobilization, timely device removal, identification of deviations from expected recovery, and discharge preparation. The ERAS Society published updated recommendations for perioperative care in elective colorectal surgery in 2025, superseding previous guidance and providing the principal contemporary framework for evidence-based perioperative management [1]. Given that successful implementation depends on the integration of multiple interventions delivered by different professional groups throughout the perioperative trajectory, an updated synthesis combining the 2025 recommendations with contemporary evidence while explicitly examining multidisciplinary contribution may provide clinically useful guidance for perioperative teams.

Aim of the review

The aim of this updated narrative review is to critically synthesize contemporary evidence regarding the multidisciplinary implementation of ERAS pathways in elective colorectal cancer surgery, drawing primarily on the 2025 ERAS Society recommendations. The review examines key perioperative components, the contributions of the healthcare professionals involved, areas in which recommendations have evolved or remain uncertain, and practical considerations for coordinated, evidence-based care. Particular emphasis is placed on preoperative optimization, patient education, prehabilitation, nutritional and hematologic optimization, bowel preparation, venous thromboembolism prevention, postoperative nutrition and mobilization, gastrointestinal recovery, discharge planning, pathway adherence, audit, and multidisciplinary implementation. Detailed anesthetic management is beyond the scope of the present review.

## Review

Methodology

This study was conducted as a structured narrative review of contemporary evidence concerning the multidisciplinary implementation of ERAS in elective colorectal surgery, with particular emphasis on colorectal cancer. The review was designed to provide a clinically focused synthesis of recent evidence rather than a systematic review, scoping review, or meta-analysis. The literature search and selection process was structured to enhance transparency and reproducibility, while no formal quantitative evidence synthesis was undertaken. Quantitative pooling was not considered appropriate because the included evidence was clinically and methodologically heterogeneous with respect to study design, ERAS components, patient populations, multidisciplinary implementation strategies, and reported clinical and implementation outcomes. For the same reason, and because the objective was a clinically focused narrative synthesis rather than estimation of a pooled intervention effect, no formal study-level risk-of-bias assessment was performed. Methodological limitations and the reported quality or certainty of evidence were instead considered when interpreting individual studies, systematic reviews, and guideline recommendations. The 2025 ERAS Society recommendations served as the principal contemporary framework, and evidence published from January 2022 through August 2026 was examined to identify recent developments, updated recommendations, and areas of uncertainty.

Eligibility criteria

Eligibility criteria were predefined according to the scope of the review and applied during the study-selection process. Eligible publications focused on adult patients undergoing elective colorectal surgery, particularly colorectal cancer surgery, and addressed ERAS pathways, multidisciplinary implementation, or predefined perioperative ERAS components. The inclusion and exclusion criteria, including population, surgical setting, interventions and ERAS components, study design, publication period, language, and scope-related exclusions, are presented in Table 1.

**Table 1:** Eligibility Criteria for Study Selection ERAS: Enhanced Recovery After Surgery; PBM: patient blood management; VTE: venous thromboembolism.

Criterion	Inclusion criteria	Exclusion criteria
Publication period	Published between January 1, 2022, and August 1, 2026	Published outside the predefined period
Language	Published in English	Not available in English
Population	Adults aged ≥18 years	Pediatric populations (<18 years)
Surgical setting	Elective colorectal surgery, with particular emphasis on colorectal cancer surgery	Emergency colorectal surgery, unless outcomes for elective surgery were reported separately
Surgical population	Colorectal surgical populations relevant to perioperative ERAS care	Exclusively non-colorectal surgical populations without direct relevance to colorectal perioperative care
ERAS relevance	ERAS pathways, multidisciplinary ERAS implementation, or individual ERAS components within the predefined scope	No clinically relevant information regarding ERAS interventions, multidisciplinary implementation, adherence, or perioperative recovery
Patient education	Preoperative patient education and counseling	—
Preoperative optimization	Preoperative optimization and risk assessment	—
Prehabilitation	Prehabilitation interventions relevant to elective colorectal surgery	—
Nutrition	Nutritional screening, optimization, and perioperative nutritional support	—
Anemia/PBM	Preoperative anemia management and patient blood management	—
Smoking/alcohol	Preoperative smoking and alcohol cessation	—
Fasting/carbohydrate loading	Preoperative fasting and carbohydrate loading	—
Bowel preparation	Mechanical bowel preparation and oral antibiotic prophylaxis	—
VTE prophylaxis	Perioperative venous thromboembolism prophylaxis	—
Postoperative nutrition	Early postoperative oral nutrition	—
Mobilization	Early mobilization and functional recovery	—
Invasive devices	Timely removal of urinary catheters and other invasive devices	—
Postoperative ileus	Prevention and management of postoperative ileus	—
Discharge/follow-up	Recovery-based discharge planning and post-discharge follow-up	—
ERAS implementation	ERAS adherence, audit, implementation, and outcome assessment	—
Multidisciplinary care	Contribution of surgeons, anesthesiologists, nurses, dietitians/clinical nutrition specialists, physiotherapists, pharmacists, stoma-care specialists, and other healthcare professionals involved in ERAS implementation	—
Study design	Clinical practice guidelines or consensus recommendations; systematic reviews and meta-analyses; randomized controlled trials; prospective or retrospective observational studies; implementation or quality-improvement studies	Case reports; small case series without relevance to ERAS recommendations or implementation; conference abstracts without sufficient methodological or outcome information; study protocols without relevant outcome data; editorials, letters, commentaries, or unsupported expert opinions
Anesthesia-specific scope	—	Studies focused exclusively on detailed anesthetic management, including anesthetic technique or depth of anesthesia, neuromuscular blockade and reversal, intraoperative hemodynamic or fluid management, or regional and neuraxial analgesic techniques
Duplicate publications	Original or otherwise eligible reports providing relevant information	Duplicate publications or duplicate reports of the same study population without additional relevant information

Information sources and search strategy

A structured literature search was conducted to identify contemporary evidence relevant to the multidisciplinary implementation of Enhanced Recovery After Surgery (ERAS) in elective colorectal surgery, with particular emphasis on colorectal cancer. PubMed/MEDLINE and Scopus were searched for English-language publications from January 1, 2022, through August 1, 2026. The search strategy combined terms related to enhanced recovery with terms related to colorectal cancer and colorectal surgery. Boolean operators, phrase searching, and database-specific field restrictions were used as appropriate. The complete search strategies and the number of records identified in each database are presented in Table 2.

**Table 2:** Database Search Strategy (January 1, 2022 to August 1, 2026) ERAS: Enhanced Recovery After Surgery; MEDI: Medicine; NURS: Nursing; MULT: Multidisciplinary; TITLE-ABS-KEY: title, abstract, and keywords.

Database	Search terms/strategy	Limits	Records
PubMed/MEDLINE	("enhanced recovery after surgery" OR ERAS OR "enhanced recovery") AND ("colorectal cancer" OR "colorectal surgery" OR "colorectal resection" OR "colon surgery" OR "rectal surgery")	English language; January 1, 2022-August 1, 2026	66
Scopus	TITLE-ABS-KEY ("enhanced recovery after surgery") AND TITLE-ABS-KEY ("colorectal cancer" OR "colorectal surgery" OR "colorectal resection" OR "colon surgery" OR "rectal surgery") AND PUBYEAR > 2021 AND PUBYEAR < 2027 AND LIMIT-TO (EXACTKEYWORD, "Human") AND LIMIT-TO (LANGUAGE, "English") AND (LIMIT-TO (SUBJAREA, "MEDI") OR LIMIT-TO (SUBJAREA, "NURS") OR LIMIT-TO (SUBJAREA, "MULT")) AND LIMIT-TO (DOCTYPE, "ar")	English language; human studies; Medicine, Nursing, or Multidisciplinary subject areas; articles; 2022-2026	464

Reference lists of relevant guidelines, systematic reviews, and meta-analyses were also examined to identify additional eligible publications. The 2025 ERAS Society recommendations served as the principal framework for defining established components and identifying areas of evolving or uncertain evidence; recent systematic reviews, meta-analyses, randomized trials, and observational/implementation studies were subsequently used to update and contextualize the evidence. A total of 530 records were identified through the database searches before duplicate removal.

Study selection and evidence prioritization

The database searches identified 530 records, comprising 66 records from PubMed/MEDLINE and 464 from Scopus. After removal of 25 duplicate records, 505 unique records remained for title and abstract screening. Screening was performed according to the predefined scope and eligibility criteria. A total of 104 records were excluded at this stage because they clearly did not meet the eligibility criteria, leaving 401 potentially relevant records.

Because the objective of the present study was to provide an updated narrative synthesis rather than a systematic review or meta-analysis, evidence selection beyond the initial structured screening was guided by relevance to the predefined ERAS components, applicability to elective colorectal surgery and colorectal cancer, methodological quality, recency, and contribution to the interpretation of contemporary multidisciplinary ERAS practice. Priority was given to current clinical practice guidelines and consensus recommendations, systematic reviews and meta-analyses, followed by randomized controlled trials and relevant observational or implementation studies. Evidence specific to colorectal cancer was prioritized over evidence derived from broader gastrointestinal surgical populations when both were available.

Following this evidence-prioritization process, 33 publications were selected for inclusion in the final narrative synthesis. The remaining potentially relevant records were not included because they provided redundant evidence, addressed ERAS components outside the predefined focus of the review, had limited applicability to contemporary elective colorectal cancer surgery, or were superseded by more recent or methodologically stronger evidence addressing the same clinical question.

Detailed anesthesia-specific studies were excluded when their primary focus was anesthetic technique, depth of anesthesia, neuromuscular blockade and reversal, intraoperative hemodynamic or fluid management, or regional and neuraxial analgesic techniques, in accordance with the predefined scope of the review. Evidence from individual publications was synthesized narratively according to the corresponding ERAS component and the contribution of the multidisciplinary perioperative team. No quantitative pooling, formal meta-analysis, or systematic risk-of-bias assessment was undertaken.

**Figure 1:**
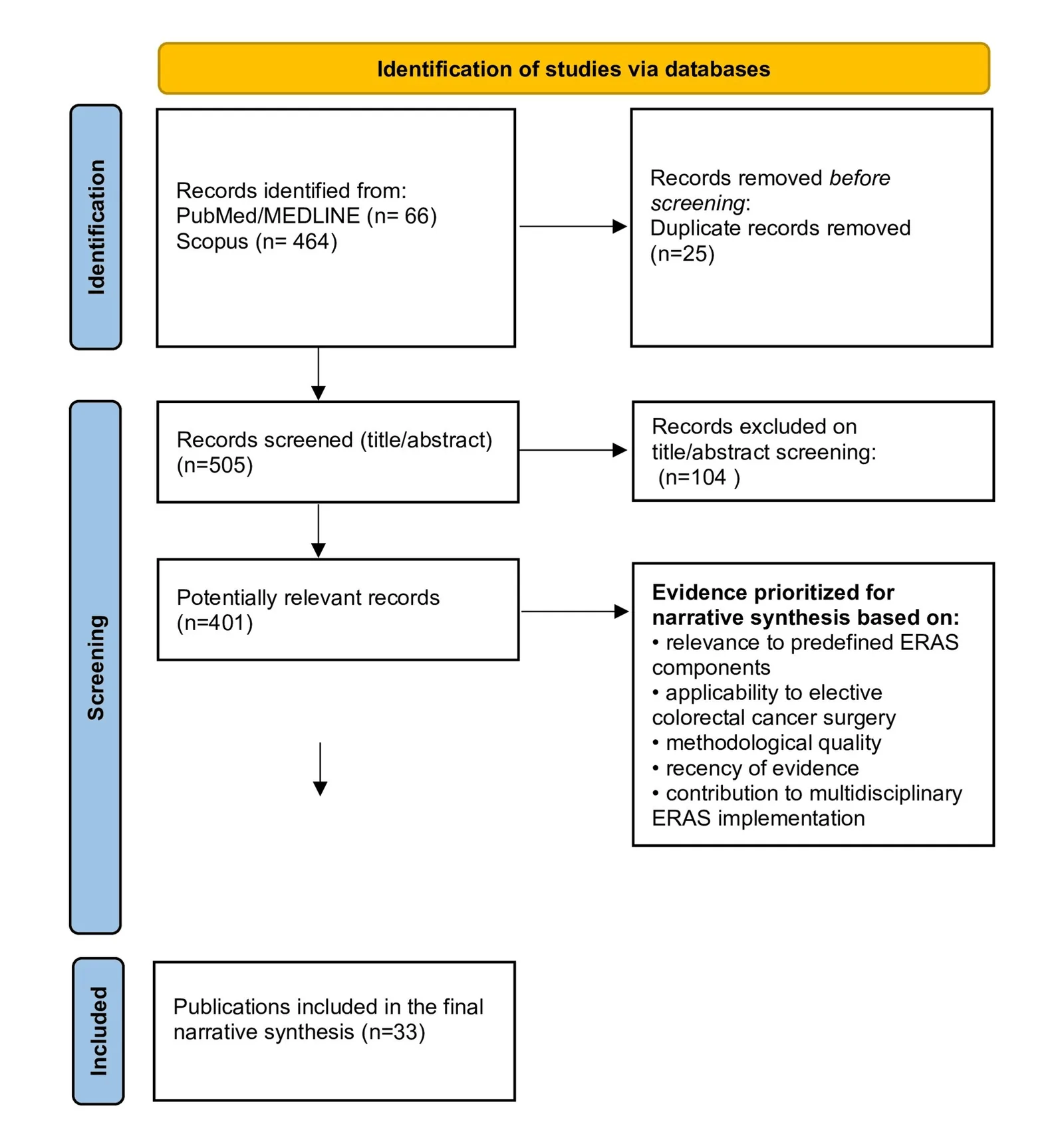
Flow Diagram of Literature Identification, Screening, and Evidence Prioritization Flow diagram of literature identification, screening, and evidence prioritization for the structured narrative review. ERAS: Enhanced Recovery After Surgery.

The literature identification and screening process is summarized in Figure 1. The flow structure was used to improve transparency in reporting database identification, duplicate removal, title and abstract screening, and subsequent evidence prioritization. It is intended as a descriptive representation of the literature-selection process and should not be interpreted as classification of the present review as a systematic review.

The multidisciplinary ERAS team

Successful ERAS implementation depends on coordinated multidisciplinary care rather than isolated interventions. The multidisciplinary team (MDT) typically includes colorectal surgeons, anesthesiologists, nurses, dietitians, physiotherapists, pharmacists, other relevant medical specialists, and allied healthcare professionals according to individual patient needs and clinical circumstances [1]. The surgeon leads surgical strategy and oncological decision-making; anesthesiology contributes to preoperative risk assessment and evidence-based anesthetic and analgesic strategies, which are beyond the scope of this review [1].

Nurses have a continuous role connecting the different phases of care, including education, symptom assessment, facilitation of early oral intake and mobilization, timely device removal, and discharge preparation [2]. A 2026 systematic review and meta-analysis of 13 studies found that ERAS pathways with identifiable nursing leadership were associated with shorter hospital stay, fewer complications, and lower readmission, without evidence of increased mortality, although heterogeneity in intervention definitions limits causal interpretation [2]. Dietitians, physiotherapists, and pharmacists contribute nutritional support, functional rehabilitation, and medication/analgesic management, respectively. Effectiveness depends not only on which interventions are included but on adherence, communication, and coordination across the team [1,2].

Physiotherapists contribute individualized functional assessment and rehabilitation, particularly for frail or functionally impaired patients, while pharmacists support medication reconciliation, antimicrobial stewardship, and multimodal analgesic strategies. Continuity across the preoperative, intraoperative, and postoperative phases depends on the multidisciplinary team's ability to adapt standardized recommendations to individual patient characteristics and recovery trajectories, rather than applying a fixed protocol uniformly to all patients.

Patient information, education, and counseling

Preoperative education should begin before admission, covering the planned procedure, ERAS principles, expected milestones, anticipated symptoms, and discharge criteria, and should address the patient's active role in recovery [1]. Education should be consistent across disciplines: surgeons address procedure-specific risks, nurses reinforce ERAS principles and assess understanding, and dietitians, physiotherapists, and stoma-care nurses provide targeted counseling [1]. Establishing realistic expectations regarding recovery milestones before surgery may facilitate engagement and adherence to postoperative components [1,3]. Preoperative counseling should discuss recovery milestones and discharge criteria rather than focusing solely on the surgical procedure itself; establishing these expectations before surgery may reduce uncertainty and facilitate adherence to postoperative ERAS components.

Education should be individualized according to health literacy, cognitive status, language, culture, comorbidities, and social support, involving family or caregivers where appropriate. For patients anticipating a stoma, structured preoperative stoma education, including site marking and practical management guidance, should be incorporated; recent evidence suggests this may reduce peristomal skin complications, although evidence for other outcomes remains of low or very low certainty [4]. Overall, preoperative education should be regarded as a structured, multidisciplinary intervention aimed at establishing realistic expectations and facilitating self-management after discharge.

Prehabilitation

Prehabilitation aims to enhance physiological and psychological capacity before surgery, and in colorectal cancer surgery may combine physical exercise, nutritional intervention, psychological support, and behavioral risk modification [5,6]. Surgeons identify candidates and ensure prehabilitation does not delay oncological treatment; physiotherapists prescribe individualized exercise; dietitians address nutritional status; and nurses coordinate education, monitoring, and adherence.

A 2023 Cochrane review concluded that prehabilitation may improve functional capacity (particularly six-minute walk test performance) but could not establish a robust effect on complications, emergency visits, or readmissions, with certainty ranging from moderate to very low [6]. In contrast, the multicenter PREHAB randomized trial found that a four-week multimodal program (exercise, nutrition, psychological support, and smoking cessation) significantly reduced severe and medical complications and improved functional recovery, although the trial was terminated prematurely due to COVID-19 [7]. A 2025 meta-analysis of 14 studies (986 patients) found improved preoperative aerobic fitness but no corresponding overall improvement in postoperative outcomes, with only one included study judged low-risk for both bias and exercise ineffectiveness [8]. A further 2025 network meta-analysis suggested multimodal prehabilitation may outperform single-component strategies for complications, physical function, and anxiety, although intervention composition varies substantially across trials [5]. Overall, the available evidence remains conflicting and heterogeneous, and the 2025 ERAS Society recommendations do not support a specific prehabilitation regimen. Although selected randomized trials and meta-analyses suggest potential benefits of multimodal prehabilitation for functional recovery and selected postoperative outcomes, the optimal components, intensity, duration, timing, and patient selection remain uncertain [1,5-8].

The multidisciplinary nature of prehabilitation is particularly relevant in colorectal cancer, since timing must be coordinated so that preoperative optimization does not delay definitive oncological treatment. Psychological support may additionally be incorporated for patients experiencing clinically relevant anxiety or distress related to diagnosis and planned treatment, reflecting the broader shift toward addressing functional, nutritional, psychological, and behavioral risk factors together rather than in isolation.

Smoking and alcohol cessation

Smoking and excessive alcohol consumption are modifiable risk factors that should be systematically assessed preoperatively [1]. The ERAS Society recommends smoking cessation at least four weeks before elective colorectal surgery, preferably supported by structured behavioral intervention combined with nicotine replacement therapy rather than brief advice alone, since cessation for less than four weeks has not consistently reduced complications [1]. A 2025 systematic review and meta-analysis found that structured preoperative smoking cessation interventions increased abstinence and reduced overall postoperative complications, although effects on wound-related complications remained uncertain and heterogeneity was considerable [9]. The four-week interval is clinically meaningful, since cessation for shorter periods before surgery has not consistently demonstrated a reduction in postoperative complications.

Alcohol consumption should likewise be assessed, with patients reporting high consumption advised to abstain for at least four weeks; this recommendation is graded weak because evidence for reductions in mortality, complications, and length of stay remains of low quality [1]. Nurses contribute to screening, education, and reinforcement, and behavioral optimization should be coordinated with the timing of oncological treatment to avoid unnecessary delay.

Nutritional screening and optimization

Malnutrition and nutritional risk should be identified early, with targeted intervention in patients at risk or with established malnutrition [1]. A 2024 systematic review and meta-analysis of seven randomized controlled trials (RCTs) (737 patients) found that preoperative oral nutritional supplements (ONS) modestly reduced overall complications, although not significant reductions in wound infection, anastomotic leakage, or urinary infection specifically, arguing against indiscriminate supplementation [10]. A broader 2024 meta-analysis across gastrointestinal malignancies (12 RCTs, 1,201 patients) similarly found fewer infectious complications and improved nutritional/inflammatory parameters with ONS, without reduction in length of stay or non-infectious complications [11].

A 2025 meta-analysis of preoperative enteral immunonutrition specific to colorectal cancer surgery suggested potential benefit for selected outcomes, but heterogeneity in formulation and protocols limits universal application [1,12]. Overall, current evidence favors individualized nutritional optimization based on risk rather than routine supplementation for all patients; nurses identify risk and monitor tolerance, while dietitians provide detailed assessment and planning. Within a multidisciplinary pathway, nutritional optimization requires coordinated assessment and follow-up: nurses contribute to early identification of risk and monitoring of intake and tolerance, dietitians provide detailed individualized planning, and surgeons integrate nutritional optimization with the timing of surgery, allowing intervention to be targeted to patients most likely to benefit rather than applied indiscriminately.

Preoperative anemia and patient blood management

Preoperative anemia is common in colorectal cancer surgery and represents a modifiable risk factor, most often related to iron deficiency from chronic blood loss, inflammation-mediated changes in iron metabolism, or reduced intake [1,13]. Screening should occur early enough to allow investigation and treatment, including hemoglobin, ferritin, and transferrin saturation, with interpretation of ferritin accounting for concurrent inflammation [13]. Management should form part of a Patient Blood Management strategy based on optimizing red cell mass, minimizing blood loss, and appropriate transfusion use [1].

Oral iron may suffice for mild anemia with sufficient time before surgery, but intravenous iron is preferred when surgery is imminent, oral therapy is poorly tolerated, or inflammation is likely to impair absorption; intravenous iron appears more effective than oral supplementation at increasing preoperative hemoglobin, although effects on transfusion requirements vary across studies [14,15]. A 2024 meta-analysis confirmed that intravenous iron significantly improves hemoglobin and reduces transfusion requirements, with heterogeneity in formulations and timing [15]. Observational 2025 data similarly reported lower transfusion requirements and shorter hospital stay with treated iron-deficiency anemia, although causality cannot be inferred [16]. Restrictive transfusion strategies are supported for most stable adults, incorporating symptoms and clinical context rather than a single hemoglobin threshold [17]. Surgeons ensure early recognition, anesthesiologists contribute blood-conservation strategies, and nurses facilitate screening and treatment adherence.

Blood transfusion should not be used routinely to correct stable preoperative anemia when alternative treatment is feasible; contemporary transfusion guidance instead supports restrictive strategies for most hemodynamically stable adults. Hematology or transfusion medicine consultation may be warranted in patients with complex anemia, unexplained abnormalities, or anticipated high transfusion requirements, underscoring that preoperative anemia should be identified and treated as early as possible within the pathway rather than accepted as an unavoidable perioperative finding.

Bowel preparation and oral antibiotic prophylaxis

Mechanical bowel preparation (MBP) alone has not consistently improved outcomes and should not be regarded as an effective infection-prevention strategy in isolation; increasing evidence instead supports combining MBP with oral antibiotics (OAB) in selected patients [1,18]. Oral antibiotics should be considered an adjunct to, not a replacement for, standard intravenous prophylaxis; regimens incorporating both are associated with lower surgical site infection (SSI) rates [18,19]. A 2025 meta-analysis of nine RCTs (3,046 patients) found that MBP+OAB significantly reduced overall SSI compared with MBP alone (RR=0.55, 95% CI: 0.44-0.68) and reduced anastomotic leakage (OR=0.45, 95% CI: 0.32-0.65), with low heterogeneity [18]. Mechanical preparation alone should therefore not be regarded as an effective infection-prevention strategy in isolation, and the distinction between colonic and rectal surgery, anastomotic level, and surgical approach remains clinically relevant when deciding whether bowel preparation is indicated.

The need for MBP varies according to procedure, anastomotic level, and approach, so bowel preparation should be incorporated into a standardized strategy rather than applied universally [1]. Surgeons determine the appropriate strategy, nurses provide instructions and monitor adherence and adverse effects (dehydration, intolerance), and pharmacists support antimicrobial stewardship. Overall, current evidence favors combining MBP with oral antibiotics as an integrated infection-prevention strategy rather than treating MBP as an independent intervention.

Preoperative fasting and carbohydrate loading

Prolonged preoperative fasting is no longer necessary for most patients. Current guidelines strongly recommend withholding solids for six hours and clear liquids for two hours before anesthesia in patients without delayed gastric emptying or aspiration risk, which is considered safe and may improve insulin sensitivity, reduce nausea/vomiting, and shorten hospital stay [1]. Carbohydrate loading has traditionally been used to attenuate postoperative insulin resistance, but the 2025 ERAS guidelines downgraded the recommendation from strong to weak, concluding that it improves insulin sensitivity without clear clinical advantage over short-fasting protocols alone [1]. This downgrade reflects the discrepancy between favorable metabolic effects and the absence of consistent benefit in major patient-important clinical outcomes. A 2026 meta-analysis of 13 RCTs (996 patients) found no significant reduction in overall complications or mortality, although secondary outcomes suggested earlier gastrointestinal recovery, earlier mobilization, shorter stay, and fewer respiratory infections, findings that require cautious interpretation given heterogeneity [20]. A further 2026 meta-analysis similarly found benefits in selected metabolic/recovery outcomes without a consistent reduction in major morbidity [21]. Importantly, short preoperative fasting and carbohydrate loading should be considered as two distinct interventions with differing strength of supporting evidence: the shift toward abbreviated fasting is well established, whereas carbohydrate-containing beverages should be regarded as a selective adjunct rather than an obligatory component applied uniformly to every colorectal ERAS pathway.

Venous thromboembolism prophylaxis

Patients undergoing colorectal cancer surgery face increased venous thromboembolism (VTE) risk from malignancy, major surgery, and immobility. Pharmacological thromboprophylaxis (typically low-molecular-weight heparin) should be used unless contraindicated, with mechanical prophylaxis and early mobilization as complementary, not substitute, measures [1]. A particularly relevant issue is whether pharmacological prophylaxis should continue after discharge, since hospital length of stay has shortened considerably while postoperative thrombotic risk may persist beyond it.

Whether prophylaxis should extend beyond discharge is particularly relevant given shortened hospital stays. A 2025 meta-analysis of five studies (2,936 patients) found extended thromboprophylaxis (≥28 days, low-molecular-weight heparin (LMWH) or direct oral anticoagulant (DOAC)-based) significantly reduced VTE without a statistically significant increase in major bleeding, although safety evidence was limited [22]. Conversely, a 2026 multicenter retrospective cohort of 2,409 patients across six ERAS-adherent centers found a 90-day symptomatic VTE incidence of only 0.2%, with no significant difference between discharge-only and extended prophylaxis, while major bleeding was more frequent with extended prophylaxis (observational design limits causal interpretation) [23]. These findings suggest that earlier mobilization and modern ERAS care may have altered baseline VTE risk compared with historical populations, although patients with prior VTE, advanced malignancy, open surgery, obesity, or major complications may still warrant extended prophylaxis. Nurses support education, subcutaneous injection teaching after discharge, and recognition of VTE/bleeding symptoms. Overall, extended prophylaxis remains supported for higher-risk patients, while a more individualized, risk-adapted approach may be appropriate for others [1,22,23].

Urinary catheter management

Prolonged catheterization increases infection risk and impedes mobilization, while excessively early removal increases urinary retention risk; contemporary practice favors early but individualized removal [1]. The 2025 ERAS guidelines recommend catheter removal within 48 hours after uncomplicated minimally invasive rectal surgery in patients without retention risk factors, balancing infection risk against the urinary dysfunction associated with pelvic dissection [1]. Systematic review evidence supports this balance: early removal after rectal surgery lowers urinary tract infection but may increase retention compared with later removal, with removal on postoperative day 3-4 previously proposed as a reasonable compromise in patients at greater risk [24].

Timing should therefore be individualized according to pelvic dissection, male sex, pre-existing urinary dysfunction, and intraoperative manipulation, avoiding routine prolonged catheterization without clinical indication. Nurses implement removal protocols, monitor bladder function, and recognize retention or infection, supporting a balance between minimizing catheter-associated infection and urinary retention. Routine prolonged catheterization should be avoided in the absence of a clinical indication. Clear multidisciplinary criteria for catheter removal and bladder assessment can reduce unwarranted variation in practice, with the objective being not simply early removal but timely removal that minimizes both catheter-associated infection and postoperative urinary retention.

Early oral feeding and postoperative nutrition

Early resumption of oral nutrition, rather than waiting for return of bowel sounds or flatus, is now standard for uncomplicated elective colorectal surgery, with intake advanced according to individual tolerance and symptoms such as nausea or distension prompting reassessment rather than routine postponement [1]. A 2024 meta-analysis of 34 RCTs found early feeding associated with earlier passage of stool and flatus, lower risk of complications (RR=0.69, 95% CI: 0.59-0.80, moderate certainty), and shorter hospital stay (low certainty), without increased vomiting risk, although substantial heterogeneity in feeding definitions and protocols limits standardization [25]. Symptoms such as nausea, vomiting, abdominal distension, or inability to tolerate intake should prompt reassessment and individualized modification of the feeding plan rather than routine postponement of nutrition.

The updated ESPEN guideline emphasizes early re-establishment of oral feeding and early nutritional therapy when risk becomes apparent; oral nutrition remains preferred, with supplements, enteral, or (when necessary) parenteral nutrition considered according to tolerance and clinical circumstances [26]. Dietitians assess requirements and progression, while nurses implement feeding, monitor tolerance, and identify patients failing to meet milestones. Overall, early oral feeding is supported by favorable effects on gastrointestinal recovery, complications, and length of stay, although optimal timing and composition remain incompletely standardized [1,25,26].

Early mobilization and functional recovery

Early mobilization aims to minimize the effects of bed rest and preserve functional capacity. The 2025 ERAS guidelines recommend mobilization beginning on the day of surgery and continuing for at least three hours daily from postoperative day 1 until discharge, although supporting evidence for this specific target is of very low quality for functional and complication outcomes and moderate quality for length of stay, so the target should be regarded as a pragmatic goal rather than a proven threshold [1].

A discrepancy exists between prescribed targets and actual activity: using wearable motion sensors, Wiesenberger et al. found that most patients did not achieve prescribed mobilization targets, and self-reported activity differed substantially from objectively measured activity, highlighting the difficulty of implementing standardized goals and the potential value of objective monitoring [27]. Barriers include pain, nausea, dizziness, catheters/lines, fear of movement, and insufficient assistance, making mobilization a multidisciplinary intervention rather than an isolated instruction. Nurses assist with initial mobilization and document milestones, while physiotherapists provide individualized rehabilitation, particularly for frail or functionally impaired patients [1,27].

Effective analgesia, appropriate fluid management, early removal of unnecessary tubes and catheters, adequate nutritional support, and patient education collectively facilitate safe progression toward functional independence. Realistic, individualized mobility goals, established jointly by surgeons, anesthesiologists, nurses, and physiotherapists, remain preferable to a uniform numerical target applied without regard for patient-specific barriers.

Prevention and management of postoperative ileus

Postoperative ileus (POI) delays gastrointestinal recovery, oral intake, mobilization, and discharge, with multifactorial pathophysiology involving surgical manipulation, inflammatory/neurogenic responses, opioid exposure, and fluid/electrolyte disturbance. Prevention emphasizes a multimodal approach: early oral feeding, opioid-sparing analgesia, appropriate fluid management, early mobilization, minimally invasive surgery, and adjuncts such as chewing gum [1]. A 2025 meta-analysis of non-pharmacological interventions (gum chewing, electroacupuncture, coffee/caffeine, abdominal massage) found several associated with earlier gastrointestinal recovery, although magnitude and consistency varied by intervention [28].

A recent meta-analysis of 30 studies (73,433 patients) reported a pooled POI prevalence of 9% (95% CI: 8%-11%), with increased risk associated with male sex (OR=2.20), operative duration >3 hours (OR=1.75), open surgery (OR=2.95), ileostomy (OR=4.78), previous abdominal surgery (OR=2.21), and age ≥65 years [29]. When recovery is delayed, management should identify reversible contributing factors and exclude mechanical obstruction, anastomotic complications, or intra-abdominal infection. Nurses systematically assess nausea, distension, tolerance, and bowel function to identify deviations early and communicate them to the team [1,28,29]. Persistent or progressive abdominal distension, nausea or vomiting, inability to tolerate oral intake, or prolonged absence of gastrointestinal function should prompt further assessment to exclude mechanical obstruction, anastomotic complications, or intra-abdominal infection, rather than being attributed by default to uncomplicated postoperative ileus.

Discharge criteria and post-discharge follow-up

Discharge should be based on achievement of predefined clinical and functional criteria (adequate oral intake, oral pain control, gastrointestinal recovery, appropriate mobilization, absence of conditions requiring continued care) rather than a fixed postoperative day; reduced length of stay is an outcome of successful recovery, not an objective in itself [1]. A 2026 meta-analysis of 13 studies (40,589 patients) found accelerated ERAS pathways were not associated with increased 30-day readmission, emergency visits, reoperation, or complications, although evidence was predominantly observational, supporting carefully selected rather than routine early discharge [30]. A 2025 meta-analysis on readmission after colorectal cancer surgery confirmed that risk is influenced by multiple patient-, disease-, and treatment-related factors, reinforcing the need for individualized transitional care [31]. Social support, health literacy, and the patient's ability to self-manage medications and postoperative care should also be considered when determining discharge readiness, since accelerated discharge should be considered only once clinical stability and functional recovery have genuinely been achieved.

Discharge planning should begin preoperatively and continue throughout hospitalization, with clear instructions on nutrition, mobilization, medications, wound and stoma care, thromboprophylaxis, and warning symptoms requiring reassessment. Post-discharge follow-up (telephone, outpatient, or other structured mechanisms) should be individualized by complexity, comorbidity, and readmission risk. Nurses assess functional readiness and coordinate discharge, while surgeons, dietitians, physiotherapists, and stoma-care specialists contribute according to need [1,30,31].

ERAS adherence, audit, and multidisciplinary implementation

ERAS effectiveness depends on consistent implementation and monitored adherence, not only on component selection [1]. In a prospective multicenter cohort across nine German hospitals, structured implementation substantially increased overall adherence across several perioperative components, demonstrating that adherence can be modified through systematic implementation rather than being fixed by patient or surgical characteristics [32]. In a 2025 observational study of 50 colorectal cancer patients, overall compliance was 78.6%, and patients achieving >80% compliance had significantly shorter mean hospital stay (5.2 vs 7.6 days, p=0.003), although the small sample and observational design limit causal inference [33].

Implementation requires clearly defined multidisciplinary responsibilities, standardized protocols, and escalation processes when recovery deviates from the expected pathway; nurses are particularly central given their continuous involvement across phases. Audit should extend beyond traditional outcomes (complications, mortality, readmission, length of stay) to adherence at individual-component and phase-specific levels, with regular feedback supporting targeted quality improvement, since successful initial implementation does not guarantee sustained adherence [1,32,33]. Continuous quality improvement is particularly important because successful initial implementation does not guarantee sustained adherence over time: ERAS pathways should be periodically reviewed against updated evidence, and local outcome and compliance data should be used to identify priorities for improvement through education, standardized documentation, and repeated audit cycles.

ERAS in high-risk patient subgroups

ERAS pathways should be applied with appropriate individualization in patients with increased perioperative risk. Relevant clinical factors may include advanced age, impaired functional recovery, malnutrition or nutritional risk, preoperative anemia, increased thromboembolic risk, and greater surgical complexity [1]. Patients undergoing open or prolonged surgery, rectal or extensive pelvic procedures, or ileostomy formation may also have a greater risk of postoperative ileus, urinary dysfunction, delayed mobilization, or other deviations from the expected recovery trajectory [24,27,29]. Accordingly, high-risk patients should not be managed through a rigid, uniform application of individual ERAS components; rather, standardized ERAS principles should be combined with individualized preoperative optimization, risk-adapted thromboprophylaxis, appropriate management of invasive devices, realistic mobilization and functional recovery targets, and structured discharge planning [1,22,23,27]. Patients with characteristics associated with increased risk of readmission may additionally require closer transitional care and post-discharge surveillance [31]. This risk-adapted approach allows multidisciplinary teams to preserve the core principles of ERAS while modifying the intensity and timing of individual interventions according to patient characteristics and surgical complexity.

Discussion and future directions

ERAS has evolved from a set of interventions into an integrated multidisciplinary model of care. Contemporary evidence supports many core components, but clinical benefit depends on coordinated delivery of multiple practices across the entire pathway together with sustained adherence, rather than any single intervention [1]. The strength of evidence is not uniform: carbohydrate loading has a less definitive effect on major outcomes than previously assumed, optimal thromboprophylaxis duration in lower-risk patients remains uncertain, and specific mobilization targets are widely adopted despite limited evidence that a particular duration independently improves major outcomes [1]. The increasing use of minimally invasive and robotic surgery, shorter hospitalization, and more structured care may also alter the baseline risks on which earlier recommendations were developed, so future studies should evaluate interventions within contemporary ERAS environments.

Protocol adherence remains a central challenge: in a prospective study of nine hospitals and 1,153 patients, adherence increased from 52% to 87% during structured implementation, accompanied by improved functional recovery and reduced length of stay, although improvement required several months, indicating that implementation is an organizational change process rather than a checklist [32]. Sustainability requires continuous education, standardized documentation, regular multidisciplinary communication, and audit feedback; higher compliance continues to be associated with shorter hospitalization in observational data, although causal interpretation remains limited [33].

The multidisciplinary dimension of ERAS deserves greater research attention, since comparatively little evidence examines how professional roles, communication, and coordination influence implementation fidelity, despite each discipline's distinct contribution. Nursing practice is particularly relevant given longitudinal involvement across the pathway, yet nursing-sensitive indicators are not consistently incorporated into ERAS research. Future studies should also move beyond length of stay as the dominant marker of success, incorporating functional recovery, symptom burden, and patient-reported outcomes, supported by digital monitoring and wearable activity devices where validated, together with greater methodological standardization of ERAS definitions, compliance thresholds, and outcome reporting.

Limitations of the review

Several limitations of this review should be acknowledged. First, although a structured literature search and predefined eligibility criteria were used, the review was designed as a narrative synthesis rather than a systematic review; therefore, evidence prioritization necessarily involved author judgment, and a formal risk-of-bias assessment was not performed. Second, the review was restricted to English-language publications published between January 1, 2022, and August 1, 2026, and relevant evidence published outside this period or in other languages may not have been captured. Third, substantial heterogeneity exists across the included literature in ERAS pathway composition, adherence definitions, surgical populations, intervention protocols, and outcome measures, limiting direct comparison between studies. Finally, evidence specifically evaluating multidisciplinary organization and individual professional contributions remains less developed than evidence evaluating individual ERAS interventions. Accordingly, conclusions regarding professional roles and implementation should be interpreted within the context of the available evidence.

## Conclusions

Enhanced Recovery After Surgery in colorectal cancer surgery should be understood as an integrated, multidisciplinary model of perioperative care rather than a collection of isolated interventions. Contemporary evidence supports coordinated implementation of preoperative optimization, appropriate perioperative management, early restoration of nutrition and mobility, prevention of postoperative complications, and recovery-based discharge planning. The effectiveness of ERAS depends not only on the selection of individual components but also on multidisciplinary coordination, consistent implementation, pathway adherence, and appropriate adaptation to patient-specific risk and surgical complexity. Surgeons, anesthesiologists, nurses, dietitians, physiotherapists, pharmacists, and other relevant specialists contribute complementary expertise across the perioperative continuum. A structured, risk-adapted, and continuously evaluated ERAS pathway may therefore provide a clinically relevant framework for improving perioperative recovery and supporting high-quality colorectal cancer care.
